# AI in Digital Pathology for Diffuse Large B-cell Lymphoma: A Systematic Review of Diagnosis and Classification

**DOI:** 10.7759/cureus.92058

**Published:** 2025-09-11

**Authors:** Jessica D Torres Luna, Basel T Tomalieh, Delvy Rebellow, Alousious Kasagga, Areeba Kabir, Karthika Murugesan, Paolo S Chavez Cavalie

**Affiliations:** 1 Pathology and Laboratory Medicine, Universidad Tecnologica Centroamericana (UNITEC), Tegucigalpa, HND; 2 Radiology, Aintree University Hospital, Liverpool, GBR; 3 Internal Medicine, Malankara Orthodox Syrian Church Medical College, Kolenchery, Kochi, IND; 4 Pathology, Peking University, Beijing, CHN; 5 Psychiatry and Behavioral Sciences, California Institute of Behavioral Neurosciences and Psychology, Fairfield, USA; 6 Psychiatry, University of North Texas, Denton, USA; 7 Surgery, Universidad Peruana de Ciencias Aplicadas, Lima, PER

**Keywords:** artificial intelligence, deep learning, diagnostic pathology, diffuse large b-cell lymphoma (dlbcl), diffuse large b lymphoma, digital pathology, machine learning

## Abstract

Diffuse large B-cell lymphoma (DLBCL) remains the most common and heterogeneous type of non-Hodgkin lymphoma. Accurate diagnosis is crucial but intensive. AI has emerged in the field as a potential to support the digital pathology workflow. This study is a systematic review of the performance and clinical utility of AI applied to digital pathology and classification of DLBCL through articles published from 2020 to 2025. We followed the Preferred Reporting Items for Systematic reviews and Meta-Analyses (PRISMA) 2020 guidelines. A total of 734 records were screened, and 11 studies met the inclusion criteria. QUADAS-2 and risk-of-bias VISualization(robvis) were used to assess bias. Data extraction included AI model architecture, diagnostic tasks, validation methods, and performance metrics. Eleven studies met the inclusion criteria, employing architectures such as convolutional neural networks, multiple instance learning, Vision Transformers, EfficientNet, U-Net, and HoVer-Net. Diagnostic metrics were consistently high: accuracies ranged from 87% to 100%, sensitivities from 90% to 100%, specificities from 52% to 100%, and area under the curve values up to 0.999. Several models outperformed pathologists in speed and precision, particularly in biomarker quantification and MYC rearrangement prediction. Risk of bias was low in index tests and reference standards, but patient selection was frequently rated as of high concern. AI-driven digital pathology demonstrates strong classification and diagnostic potential for DLBCL, achieving high accuracy across diverse methods and datasets. However, selection bias, limited external validation, and lack of standardization remain barriers. Continued research with multicenter, prospective validation is needed before routine clinical integration. Further research and standardization are needed for broader clinical integration in the field of digital pathology.

## Introduction and background

According to the United States Cancer Registry data, diffuse large B-cell lymphomas (DLBCLs) have an adjusted incidence rate by age of 7.2 per 100,000 annually [1]. As the most common subtype of non-Hodgkin lymphoma, DLBCL represents a heterogeneous group of aggressive lymphoproliferative disorders characterized by large cells that infiltrate and replace normal lymphatic tissue. These cells display a diffuse growth pattern in which their nuclear size may equal or surpass that of a normal macrophage or be twice the size of a typical lymphocyte. DLBCL can arise de novo or evolve from a lower-grade lymphoma. It displays a spectrum of cytological morphology, where the 2022 WHO classification prioritizes genetic and molecular profiling based on cell of origin, site of involvement, and hallmark mutations [2].

These subtypes include DLBCL, not otherwise specified, which serves as the default category; high-grade B-cell lymphoma with MYC and BCL2 and/or BCL6 rearrangements, characterized by genetic abnormalities that drive more aggressive disease; primary CNS or testicular DLBCL, defined by their location; Epstein-Barr virus-positive DLBCL, associated with chronic viral infection; and other less common site- or pathogen-specific variants [3]. Among NHL, DLBCL remains the most frequently diagnosed subtype, accounting for 30-40% of B-cell NHLs. Its incidence increases with advancing age and demonstrates a higher prevalence within the male demographic [4].

For DLBCL, pathologists use the evaluation of H&E-stained tissue sections, accompanied by immunohistochemistry (IHC) and, in other instances, flow cytometry to improve its diagnostic accuracy. Fluorescence in situ hybridization (FISH), a molecular test used to detect chromosomal rearrangements, is also used in combination, although it can be time-consuming and expensive. In practice, diagnosing DLBCL can be challenging due to its great heterogeneity, and it must be successfully distinguished from other types of lymphomas to aid patient treatment [5,6].

AI-based tools hold the potential to enhance diagnostic precision and operational effectiveness by assisting pathologists in the analysis of digitized slides. These instruments can enhance consistency and reduce workload for pathologists. Recent investigations have shown that machine learning and deep-learning methodologies can alleviate labor demand while preserving their performance with digital slides [7]. The potential of AI tools has been explored on tasks ranging from slide-level lymphoma detection to detailed subtype classification and prognostication [8,9]. Additional research has identified that AI algorithms can classify numerous lymphoma subtypes within a singular model and predict underlying tumor biomarkers derived from standard H&E morphology [7].

Despite very promising results, the implementation of AI, particularly in DLBCL pathology, remains in the primary stages. A considerable number of studies have been published, but their risk of bias is still being studied. This highlights the need for a synthesis of evidence to document the process and challenges of these instruments. Therefore, we have embarked on this systematic review to assess the current state of AI-driven digital pathology in relation to the diagnosis and classification of DLCBCL. By analyzing studies published from 2020 to 2025, we aim to synthesize the performance of AI applications, assess their clinical effectiveness, and identify limitations that will aid future research. This systematic review will highlight the contributions of new-age machine learning in improving diagnosis and patient care.

## Review

Methods

Guidelines

This study was conducted in accordance with the Preferred Reporting Items for Systematic reviews and Meta-Analyses (PRISMA) 2020 statement [10]. The selection process was done utilizing a PRISMA flow diagram [11]. The risk of bias of the individual studies was assessed using the QUADAS-2 tool [12]. Visual representations of bias were generated utilizing the risk of bias visualization (robvis) tool [13]. All methods were established prior to data extraction and registered in PROSPERO for review protocol in (registration ID: CRD420251075386). The PICO question was structured as follows: Population (P): Human patients undergoing histological evaluation for DLBCL, Intervention (I): application of AI in digital pathology, Comparison(C): Traditional pathologist interpretation or manual diagnostic methods, or no direct comparator when AI performance was reported independently, and Outcome (O): Diagnostic or classification performance of AI models.

Search Sources and Search Strategy

Databases utilized for the search were PubMed, Scopus, and Google Scholar. These databases were searched to capture relevant literature, and the established inclusion and exclusion criteria were applied uniformly to all retrieved records. Other databases (e.g., Embase, Web of Science, and Cochrane Library) were not incorporated due to overlap with PubMed and Scopus and the narrow focus of this review. The keywords consisted of “Lymphoma”, “Large B-cell lymphoma”, “machine learning”, “Artificial intelligence”, and “Digital pathology”. The search strategy also incorporated MeSH terms and Booleans shown in Table 1.

**Table 1:** Summary of search strategy across databases and number of articles retrieved CNN: convolutional neural network; DLBCL: diffuse large B-cell lymphoma; IHC: immunohistochemistry; MeSH: Medical Subject Headings

Database	Keywords/search strategy	Results (n)
Google Scholar	("diffuse large b-cell lymphoma" OR "DLBCL") AND ("artificial intelligence" OR "machine learning" OR "deep learning" OR CNN) AND ("digital pathology" OR "Whole-slide imaging" OR histopathology OR H&E OR IHC) AND (diagnosis OR detect* OR classif*)	488
PubMed	(("lymphoma, large b-cell, diffuse"[MeSH Terms] OR "diffuse large b-cell lymphoma"[All Fields] OR DLBCL[tiab]) AND ("artificial intelligence"[MeSH Terms] OR "machine learning"[MeSH Terms] OR "deep learning"[All Fields] OR CNN[tiab]) AND ("digital pathology"[MeSH Terms] OR "Whole-slide imaging"[All Fields] OR histopathology[All Fields] OR H&E[All Fields] OR IHC[All Fields]) AND (diagnosis[tiab] OR detect*[tiab] OR classif*[tiab])) AND ("2020/01/01"[PDat] : "2025/06/16"[PDat])	103
Scopus	(TITLE-ABS-KEY("diffuse large b-cell lymphoma" OR DLBCL) AND TITLE-ABS-KEY("artificial intelligence" OR "machine learning" OR "deep learning" OR CNN) AND TITLE-ABS-KEY("digital pathology" OR "Whole-slide imaging" OR histopathology OR H&E OR IHC) AND TITLE-ABS-KEY(diagnosis OR detect* OR classif*)) AND PUBYEAR > 2019	143
Total of articles identified (n)		734
Total of articles after removing duplicates (n)		108

Eligibility: Inclusion and Exclusion Criteria

We included studies that examined the application of AI in digital pathology for the diagnosis or classification of DLBCL using human tissue samples. Eligible studies met the following criteria: involved human subjects, applied AI models for DLBCL diagnosis or classification, utilized whole-slide imaging of histopathological data, were published between 2020 and 2025, and were written in English or Spanish. The restriction to these two languages reflects the working languages of the review team, allowing accurate interpretation of study findings while minimizing the risk of misclassification or translation errors. Only studies that reported diagnostic outcomes were eligible; purely prognostic or treatment-prediction studies were excluded. Studies that reported diagnostic outcomes in addition to prognostic analyses were eligible.

Exclusion criteria encompassed animal studies, investigations limited to non-DLBCL hematologic malignancies, prognostic-only analyses, and treatment prediction models. We also excluded non-original publications such as conference abstracts, editorials, opinion pieces, and grey literature. Articles published outside the specified five-year period were not considered.

Selection Process and Data Collection Process

Two reviewers independently screened for titles and abstracts based on the eligibility criteria. Disagreements were resolved by consensus; if consensus could not be reached, a third reviewer was available for arbitration. Full-text articles were retrieved and assessed for the inclusion criteria. Inconsistencies were checked by discussion among the reviewers. Duplicate records were eliminated using EndNote Basic [14]. No automation tools were used during the screening and selection process.

Data was extracted into standardized tables with the following items: first author, year of publication, study design, AI model used, image type used, dataset size, diagnostic focus, and if the study compared results with pathologists' performance. Diagnostic performance metrics, model validation type, and summary tables or figures with the data presented were also included. The reviewers discussed missing or unclear data.

Risk of Bias Assessment

Risk of bias was assessed independently by two reviewers using the QUADAS-2 tool [12]. This tool evaluated four items: patient selection, index test, reference standard, and flow and timing. The results were categorized as low risk, high risk, some concerns, or no information. Visual representations of risk of bias were generated using the robvis tool [13].

Results

Study Selection

**Figure 1:**
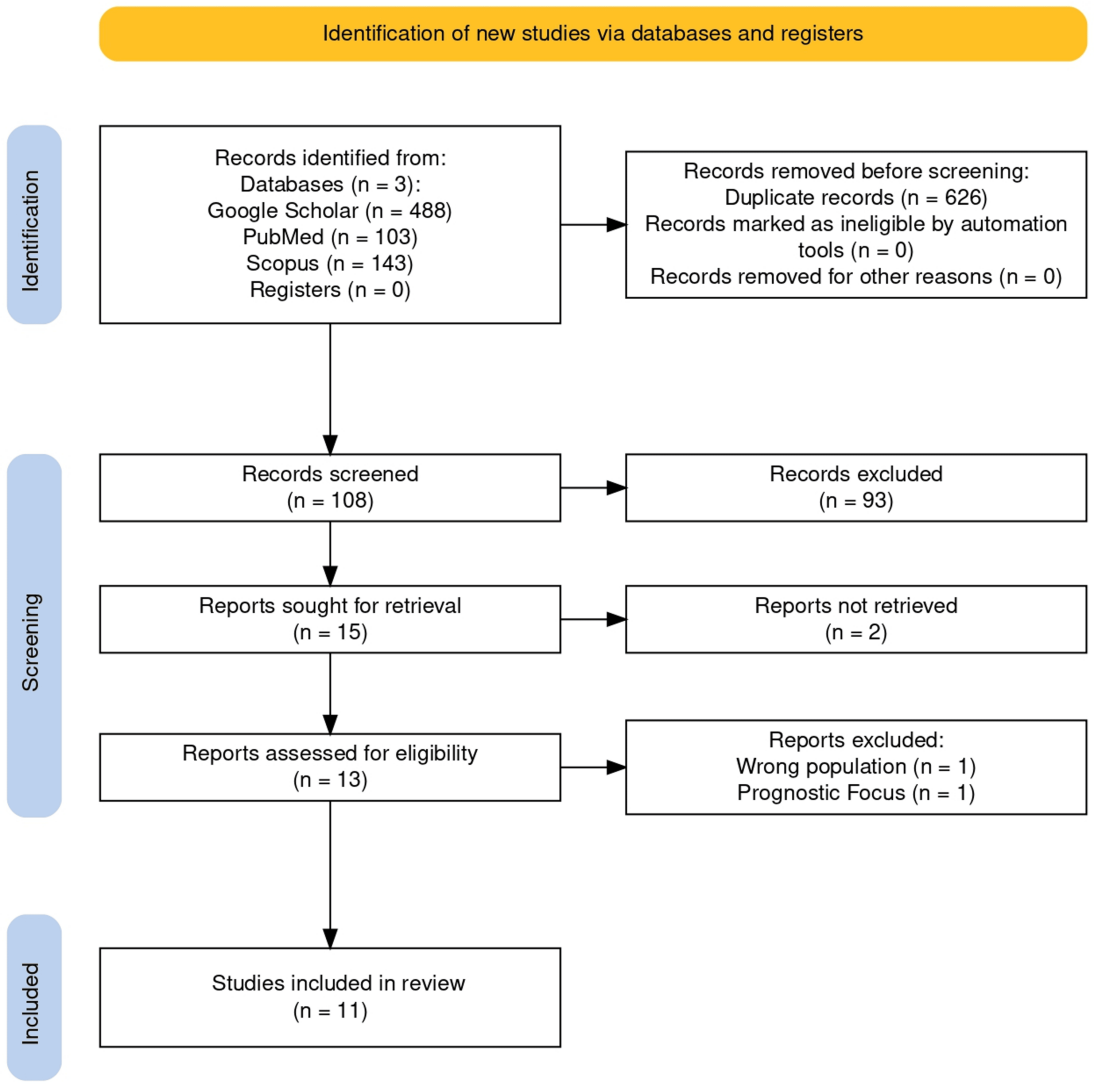
PRISMA flow diagram PRISMA: Preferred Reporting Items for Systematic reviews and Meta-Analyses

A total of 734 articles were extracted across the three databases (Google Scholar, PubMed, and Scopus) following the established search strategy. After the removal of duplicates using EndNote Basic, a total of 108 articles remained for screening. Titles and abstracts were independently reviewed by the researchers based on the inclusion and exclusion criteria. Fifteen studies remained for full-text assessment; however, two could not be retrieved due to closed access, leaving 13 articles to be assessed. Their absence may represent a potential source of bias in the review. Two additional studies were excluded after full-text screening due to a prognostic-only focus, and others for lacking a specific analysis of DLBCL diagnosis. Eleven studies were included in the final analysis for this study. The selection process is detailed in Figure 1, following the PRISMA flow diagram guidelines. Reasons for full-text exclusions are provided in Appendix A.

Study Characteristics

The 11 studies that met the inclusion and exclusion criteria were analyzed in this systematic review study. Across the studies, they consisted of diverse sample sizes, some using cohorts or biopsy samples, while others utilized larger datasets. The studies’ DLBCL samples came from two geographic regions (North America and Asia). A wide range of histological preparations was used, such as H&E-stained slides, IHC staining, and formalin-fixed paraffin-embedded tissues. The AI approaches in the studies varied; they included deep learning architectures such as convolutional neural networks (CNNs), a convolutional architecture for biomedical image segmentation (U-Net), a CNN architecture for performance and efficiency (EfficientNet), a horizontal and vertical residual network for nuclear segmentation and classification (HoVer-Net), and attention-based multiple instance learning (AB-MIL). Other studies also incorporated transfer learning, self-supervised learning, or hierarchical classification systems that mimic pathologists' diagnostic workflows.

For reference standards, some studies compared AI outcomes to pathologists' annotations or diagnoses based on IHC, FISH, or molecular testing. In studies without direct comparison to pathologists, the AI models served as tools for assistance, reducing observer variability and easing diagnostic workflows. Several AI models aimed to automate cell quantification, highlight diagnostic regions, and offer rapid pre-screening to flag heterogeneous cases. A summary of study characteristics is presented in Table 2.

**Table 2:** Characteristics of included studies evaluating AI applications in DLBCL AB-MIL: attention-based multiple instance learning; AuxCNN: auxiliary convolutional neural network; BCL2: B-cell lymphoma 2; BCL6: B-cell lymphoma 6; CD20: cluster of differentiation 20; CNN: convolutional neural network; DLBCL: diffuse large B-cell lymphoma; DHL/THL: double-hit/triple-hit lymphoma; DT: decision tree; EBER: Epstein-Barr virus-encoded RNA; FFPE: formalin-fixed paraffin-embedded; FISH: fluorescence in situ hybridization; FL: follicular lymphoma; GCB: germinal center B-cell; HGBL: high-grade B-cell lymphoma; HoLy-Net: H&E/IHC-adapted HoVer-Net; HoVer-Net: horizontal and vertical residual network; IHC: immunohistochemistry; k-NN: k-nearest neighbor; LME: lymphoma microenvironment; MALT: mucosa-associated lymphoid tissue; MIL: multiple instance learning; MLP: multi-layer perceptron; MYC: myelocytomatosis oncogene; NL: normal lymph node; PD-L1: programmed death-ligand 1; ResNet: residual neural network; RF: random forest; RL: reactive lymphoid hyperplasia; ROI: region of interest; RPART: recursive partitioning and regression trees; SAM: Segment Anything Model; scRNA-seq: single-cell RNA sequencing; SDL: stochastic diagonal learning; SVM: support vector machine; TMA: tissue microarray; ViT: Vision Transformer; WSI: whole-slide image

Author and year	Study design	AI model used	Image type	Dataset size	Diagnostic focus	Comparison with a pathologist
Li et al., 2020 [6]	Retrospective multi-center diagnostic classification study	GOTDP-MP-CNNs (ensemble of 17 CNNs + global optimization via SDL)	H&E-stained FFPE slide images at 400× (captured via scanner or camera)	1005 images (Hosp A) + 402 images (Hosp C) + 3123 square images (Hosp B) + 531 test images	Binary classification: DLBCL vs. non-DLBCL (high-accuracy DL aid for diagnosis)	Yes
Miyoshi et al., 2020 [15]	Retrospective diagnostic study with cross-validation against pathologist diagnoses	Custom 11-layer CNN	H&E WSIs at 5x, 20x, 40x	388 cases (259 DLBCL, 89 FL, 40 RL)	Multi-class classification of DLBCL vs. FL vs. RL	Yes
Naji et al., 2024 [16]	Retrospective segmentation study	HoLy-Net (optimized HoVer-Net); compared with Mask R-CNN	H&E and IHC-stained WSIs at 40×	152,795 annotated nuclei from 700+ images across 74 patients (LyNSeC dataset)	Segmentation of neoplastic nuclei in DLBCL to enable immune profiling and morphological quantification	No
Perry et al., 2023 [17]	Retrospective diagnostic classification study	Deep learning with self-supervised pretraining + MIL	H&E-stained WSIs (scanned at 40×, analyzed at 20×)	57 biopsies from 55 patients (32 train and 25 validation)	Detection of DHL/THL among high-grade B-cell lymphomas (DLBCL and HGBL)	No
Swiderska-Chadaj et al., 2021 [18]	Retrospective diagnostic classification study	U-Net for likelihood mapping + Random Forest for WSI classification	H&E-stained WSIs (scanned at 20x) ± CD20 IHC reference	287 slides (245 internal: 140 train, 31 tune, 74 validate; 42 external)	Prediction of MYC rearrangement in DLBCL to reduce reliance on FISH testing	Yes
Tavolara et al., 2024 [19]	Translational study using the AB-MIL model trained on TMAs and validated on WSIs	AB-MIL + ResNet50 feature extractor	IHC-stained TMAs and WSIs (c-MYC and BCL2); 40×	378 TMA cores (train); 52 c-MYC and 56 BCL2 WSIs	Automated quantification of c-MYC and BCL2 expression for double-expressor stratification in DLBCL	Yes
Vrabac et al., 2021 [20]	Dataset descriptor study with prognostic morphological feature analysis	HoVer-Net (ResNet-50 backbone)	H&E- and IHC-stained TMAs (digitized at 40×	42 TMA slides from 209 DLBCL patients; survival data from 170 H&E cases	Prognostic morphometric analysis of segmented nuclei in DLBCL to predict survival outcomes	No
Wang et al., 2025 [21]	Retrospective digital pathology and algorithm development study for LME classification	QuPath-based quantifiers + RPART decision tree + k-NN + Seurat/Harmony (scRNA-seq)	H&E and IHC-stained WSIs (Aperio GT 450; bright-field)	682 DLBCL patients (315 with stained tissue: 190 train, 125 test)	Classification of DLBCL LME subtypes for prognosis and immunotherapy guidance	No
Xu et al., 2024 [22]	Multicenter retrospective observational study using IHC marker data	Logistic Regression, SVM, MLP, DT, RF, XGBoost	IHC marker profiles + EBER (structured data)	14,927 patients (8,808 internal, 6,119 external) across five classification tasks	Hierarchical classification of lymphoma subtypes, including DLBCL	No
Yamaguchi et al., 2025 [23]	Retrospective multi-class classification study	EfficientNet-B0 (ImageNet pretrained); compared with AlexNet, VGG16, and ResNet18	H&E WSIs grayscale patch images (224×224 px)	160 patients; 11,073 training + 962 test patches	Multi-class classification of lymphoma subtypes (NL, MALT, GCB/non-GCB DLBCL) and Ki67-based risk stratification	No
Yan et al., 2024 [24]	Retrospective diagnostic validation study	ViT for ROI segmentation + AuxCNN for cell detection + NuClick & SAM for cell segmentation	PD-L1 IHC WSIs at 40×	220 internal + 61 external; 146,439 annotated cells	Quantitative assessment of PD-L1 expression in DLBCL via AI-based TPS analysis	Yes

Diverse validation strategies (internal, external, and cross-validation) were performed across the 11 studies to assess model applicability. We documented the diagnostic performance metrics reported by each study in Table 3. These include accuracy, sensitivity, specificity, F1-score, precision, recall, and area under the curve (AUC); task-specific measures like intraclass correlation coefficient (ICC) and concordance index (C-index) were also documented. We also included a “location in article” column in which each study’s metrics could be identified. In cases where values were not reported, they are included as “NR” in the table.

**Table 3:** Validation strategies and diagnostic metrics of included studies evaluating AI applications in DLBCL AUC: area under the curve; DLBCL: diffuse large B-cell lymphoma; Ext: external validation; F1-score: harmonic mean of precision and recall; FL: follicular lymphoma; ICC: intraclass correlation coefficient; IHC: immunohistochemistry; Int: internal validation; NR: not reported; Prec: precision; RL: reactive lymphoid hyperplasia; ROC: receiver operating characteristic; WSI: whole-slide image

Study	Validation type(s)	AUC	Accuracy	Sensitivity	Specificity	F1-score/precision/recall	Location in article
Li et al., 2020 [6]	Internal + external	NR	100% (Hosp A, C); 99.71% (Hosp B)	100%	100%	NR	Page 5
Miyoshi et al., 2020 [15]	5-fold cross-validation	DLBCL: 1.00, FL: 0.99, RL: 1.00	Cross-val: 87-94%, Ensemble: 97%	DLBCL: 1.00, FL: 0.941, RL: 0.905	Inferred from ROC/Conf Matrices	DLBCL F1: 0.984, Prec: 0.969	Page 8
Naji et al., 2024 [16]	5-fold cross-validation	NR	0.923 (H&E), 0.964 (IHC)	NR	NR	F1: 0.876 (H&E), 0.895 (IHC)	Page 8
Perry et al., 2023 [17]	Internal (test set)	0.95	92%	100%	87%	NR	Page 7
Swiderska-Chadaj et al., 2021 [18]	Internal + external	0.74 (Int), 0.83 (Ext)	NR	90-95%	52-53%	NR	Page 3
Tavolara et al., 2024 [19]	10-fold cross-val + external	NR	NR	MYC: 0.743-0.857, BCL2: 0.706-0.938	MYC: 0.930-0.991, BCL2: 0.690-0.951	NR	Page 5
Vrabac et al., 2021 [20]	Internal (Bootstrap)	NR	NR	NR	NR	C-index = 0.700 (95% CI: 0.651-0.744)	Page 5
Wang et al., 2025 [21]	Internal + external + biological	>0.80 (marker level)	Concordance: 83.7% (train), 81.6% (test)	NR	NR	NR	Page 7
Xu et al., 2024 [22]	Internal + temporal + external	0.96 (DLBCL subtype)	0.931 (Ext, DLBCL subtype)	Macro-Recall: up to 0.874	NR	Macro-F1: up to 0.880	Page 8
Yamaguchi et al., 2025 [23]	K-fold cross-validation	3-class: 0.999, 4-class: 0.86	>0.94 (2-class), >0.90 (3-class)	>0.92 (2-class)	0.84-0.89	>0.92	Pages 5 and 7
Yan et al., 2024 [24]	Internal + external	NR	NR	NR	NR	ICC = 0.96 (95% CI: 0.94-0.97)	Pages 5 and 6

Risk of Bias in Studies

**Figure 2:**
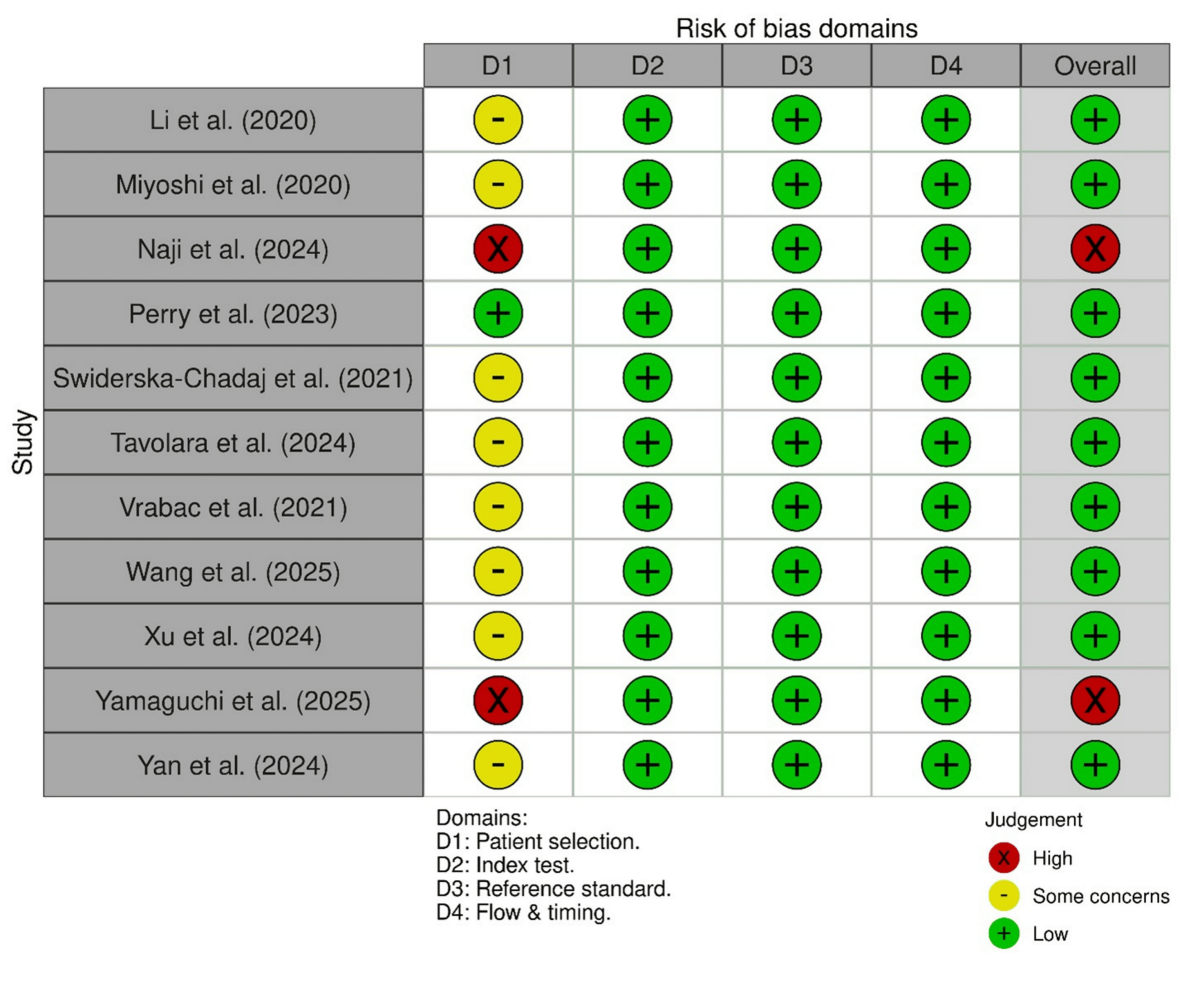
QUADAS 2 traffic light plot [6,15-24]

**Figure 3:**
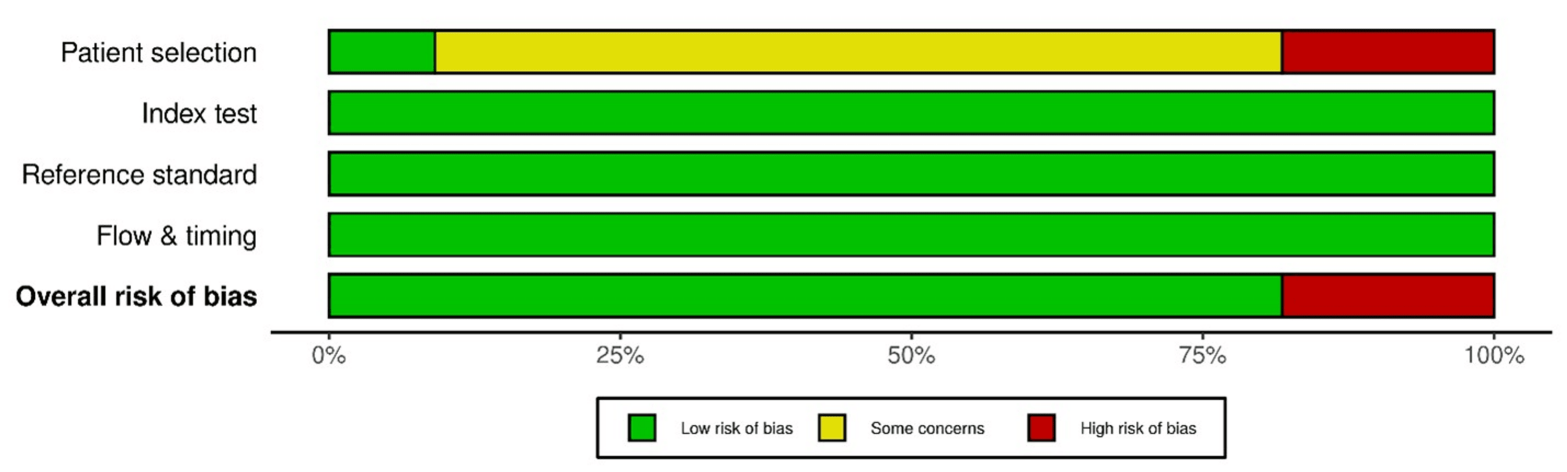
QUADAS 2 summary bar plot

Risk of bias was assessed using the QUADAS-2 tool, and figures demonstrating risk of bias were generated using the robvis platform. Most studies reported low risk of bias across most items: index test (D2), reference standard (D3), and flow and timing (D4). In contrast, patient selection (D1) had some concerns due to retrospective data collection, unclear inclusion or exclusion criteria, or convenience sampling from digital pathology archives. This limits the generalizability of model performance to clinical settings. Naji et al. and Yamaguchi et al. were rated as high risk due to concerns in patient selection, and reliance on pre-annotated datasets without the description of the original case sourcing added to the high-risk judgement [16,23]. The reference standards used in all studies were considered acceptable, but there was a lack of blinding, which was considered a limitation. Figure 2 and Figure 3 are a visual summary of the domains used in the QUADAS 2 tool assessment generated by using robvis.

Results of Synthesis

Across the 11 studies, several models were designed to do binary or multi-class classification tasks, segmentation, and biomarker quantification, and underwent external or internal validation utilizing histopathological data. Their performance metrics (AUC, accuracy, sensitivity, specificity, F1-score, etc.) showed increased performance compared to those of pathologists, as observed in studies from Li et al. [6], Miyoshi et al. [15], Swiderska-Chadaj et al. [18], Tavolara et al. [19], and Yan et al. [24].

Li et al. evaluated a multi-center CNN ensemble model, which achieved diagnostic accuracy of 99.71%-100% across hospitals and 100% sensitivity and specificity in the DLBCL tissue vs. the non-DLBCL tissue differential [6]. Miyoshi et al. also developed a CNN classifier for differentiating DLBCL, follicular lymphoma, and reactive lymphoid hyperplasia, reporting AUC values of 1.00 and an ensemble accuracy of 97% [15]. The F1-score of 0.984 highlighted the model’s strength in analyzing differential diagnosis.

Naji et al. utilized HoLy-Net for segmenting neoplastic nuclei, achieving an F1-score of 0.876-0.895 on both H&E and IHC images, making the downstream morphometric and immune cell analysis possible [16]. Perry et al. applied a self-supervised MIL-based model to identify double and triple hit lymphomas within high-grade B cell lymphomas, achieving 92% accuracy, 100% sensitivity, and 87% specificity without supplementary testing [17].

Swiderska et al. (2021) established a U-Net-based model to predict MYC rearrangements using H&E images [18]. The model demonstrated AUC values of 0.74 internal and 0.83 external, with sensitivity values between 90 and 95%, performing better than MYC IHC for FISH triage purposes. Tavolara et al. used an AB-MIL model to automate the quantification of c-MYC and BCL2 IHC expression, which correlated with pathologist assessments and improved prognostic stratification of double-expressor DLBCL, a subtype of DLBCL [19].

Vrabac et al. used HoVer-Net to extract morphometric features in DLBCL and correlated them with survival data [20]. The model achieved a prognostic C-index of 0.700, indicating a moderate prognostic value. Wang et al. developed a machine learning pipeline mixing histology and single-cell RNA sequencing to classify DLBCL lymphoma microenvironments [21]. The model demonstrated >80% AUC and maintained strong concordance (81.6-83.7%) across internal and external validation sets.

Xu et al. applied six classification algorithms on IHC marker data for hierarchical classification of lymphoma subtypes, including DLBCL [22]. Their models reported an AUC of 0.96 for DLBCL subtype identification and external accuracy of 93.1%, with Macro-F1 scores reaching 0.880, demonstrating malleability in structured data contexts. Yamaguchi et al. (2025) evaluated EfficientNet-B0 for multiclass lymphoma classification and Ki-67-based risk stratification [23]. The model achieved an AUC of 0.999 and an accuracy of 90%, with strong precision and subtype recognition.

Finally, Yan. et al used a vision transformer (ViT)-based segmentation model with auxiliary CNNs to assess PD-L1 expression in DLBCL [24]. The model achieved an ICC of 0.96 compared with manual scoring by pathologists, demonstrating high reproducibility and great potential as a digital tool for identifying DLBCL patients for targeted PD-L1 expression.

Certainty of Evidence

A comprehensive quantitative meta-analysis could not be performed because of the great amount of methodological diversity found among the studies reviewed in this analysis. The AI models exhibited variability in architecture (e.g., CNNS, U-Net, EfficientNet, HoVer-Net, and AB-MIL), diagnostic aims, imaging techniques (H&E, IHC, and WSI), and metrics for performance evaluation. Therefore, a narrative synthesis was employed to report findings across various applications of AI. Most studies indicated a high diagnostic accuracy, frequently surpassing 90% and infrequent instances either exceeding or closely aligning with the performance of pathologists. AI proved to be skillful in tasks related to automated scoring, differentiation of lymphoma subtypes, and preliminary screening of genetic anomalies. Studies using external validation cohorts further documented the potential for generalizability of these models across different institutions and datasets. The certainty of evidence was not systematically assessed using the GRADE framework, as we reviewed studies concerning diagnostic test accuracy that involve AI, in which the outcome measures and model tasks are not directly comparable.

However, the consistency of results across the 11 studies implies a moderate to high degree of confidence in the diagnostic capabilities of AI within the context of digital pathology for DLBCL. Future research should evaluate prospective validation in their studies, as well as standardization of performance metric reporting and transparency of model development to fortify the evidence base.

Discussion

This systematic review suggests that AI models show high diagnostic potential in the histopathological evaluation of DLBCL. Diverse deep learning designs like CNNs, ViTs, and MIL demonstrated high diagnostic metrics and reliability across their implementation tasks. These findings are consistent with the literature in digital pathology, where AI continues to be evaluated for its variability and support to workflow optimization in diagnostic hemopathology [7]. In comparison with broader reviews such as Fu et al. and Bai et al., this review contributes to the present literature by focusing specifically on DLBCL, a highly heterogeneous lymphoma, applying PRISMA and QUADAS-2 methodology, and synthesizing diagnostic performance across available studies [5,9]. By consolidating evidence on AI tools in DLBCL, this review underscores their potential role in automating difficult diagnostic tasks and guiding future efforts toward safe integration into clinical practice.

Li et al. and Miyoshi et al. reported that CNN-based models achieved very high performance in DLBCL classification, including perfect accuracy and AUCs within their datasets [6,15]. Yan et al. further supported AI’s reliability, achieving strong agreement with pathologists’ scoring, indicating promise in biomarker quantification tasks [18]. Swiderska et al. used AI to predict MYC rearrangements based on H&E morphology, reducing the need for FISH testing, while Tavolara et al. quantified c-MYC and BCL2 expression with AI, enabling prognostic stratification in double-expressor lymphomas [19,24].

Furthermore, the diagnostic and biomarker studies expanded the models’ scope toward prognostic and microenvironmental applications. For example, Perry et al. applied a MIL-based model to detect double- and triple-hit lymphomas, while Wang et al. integrated histopathology and transcriptomics to classify DLBCL lymphoma microenvironments, reporting high AUC and validation concordance [17,21]. Xu et al. applied IHC marker data in a hierarchical model, achieving strong external accuracy, while Yamaguchi et al. used EfficientNet to stratify lymphoma subtypes and risk, with AUCs nearing 1.0 and accuracy greater than 90%. Together, these studies illustrate how AI tools may evolve into more comprehensive decision-support systems [22].

As shown in Table 3, several studies reported exceptionally high performance, in some cases approaching 100% accuracy. However, many of these results were derived from internal or cross-validation on relatively small or homogeneous datasets, raising the possibility of overfitting and inflated performance estimates. Only a limited number of studies, most notably Swiderska et al. [18] and Wang et al. [21], incorporated external or multicenter validation, which is essential to demonstrate generalizability across institutions, staining protocols, and patient populations. In addition, heterogeneity in the study designs and outcome measures complicates direct comparisons of model performance.

Practical barriers also limit translation into everyday practice. Differences in slide preparation, staining protocols, and scanner technology could reduce reproducibility when models are applied outside their development setting. Several studies, including Naji et al. [16] and Yamaguchi et al. [23], relied heavily on pre-annotated datasets, which may not fully capture the complexity of real-world diagnostic protocols. Diagnostic metrics were reported inconsistently across studies, with some emphasizing accuracy and AUC, while others focused on F1-scores or ICC, making cross-comparisons challenging and highlighting the need for standardized reporting frameworks.

AI-assisted pathology tools for DLBCL appear promising for enhancing diagnostic workflows, reproducibility, and efficiency. CNN classifiers for morphological diagnosis [6,15] and biomarker quantification models [19,24] appear promising for enhancing diagnostic workflows, reproducibility, and efficiency. This holds potential value in both resource-rich and high-volume settings. It is worth noting, however, that such systems should be viewed as complementing rather than replacing pathologists. While these models can outperform humans in narrow classification or quantification tasks, pathologists integrate broader contextual information such as IHC, molecular findings, and clinical history, which current algorithms cannot replicate.

**Figure 4:**
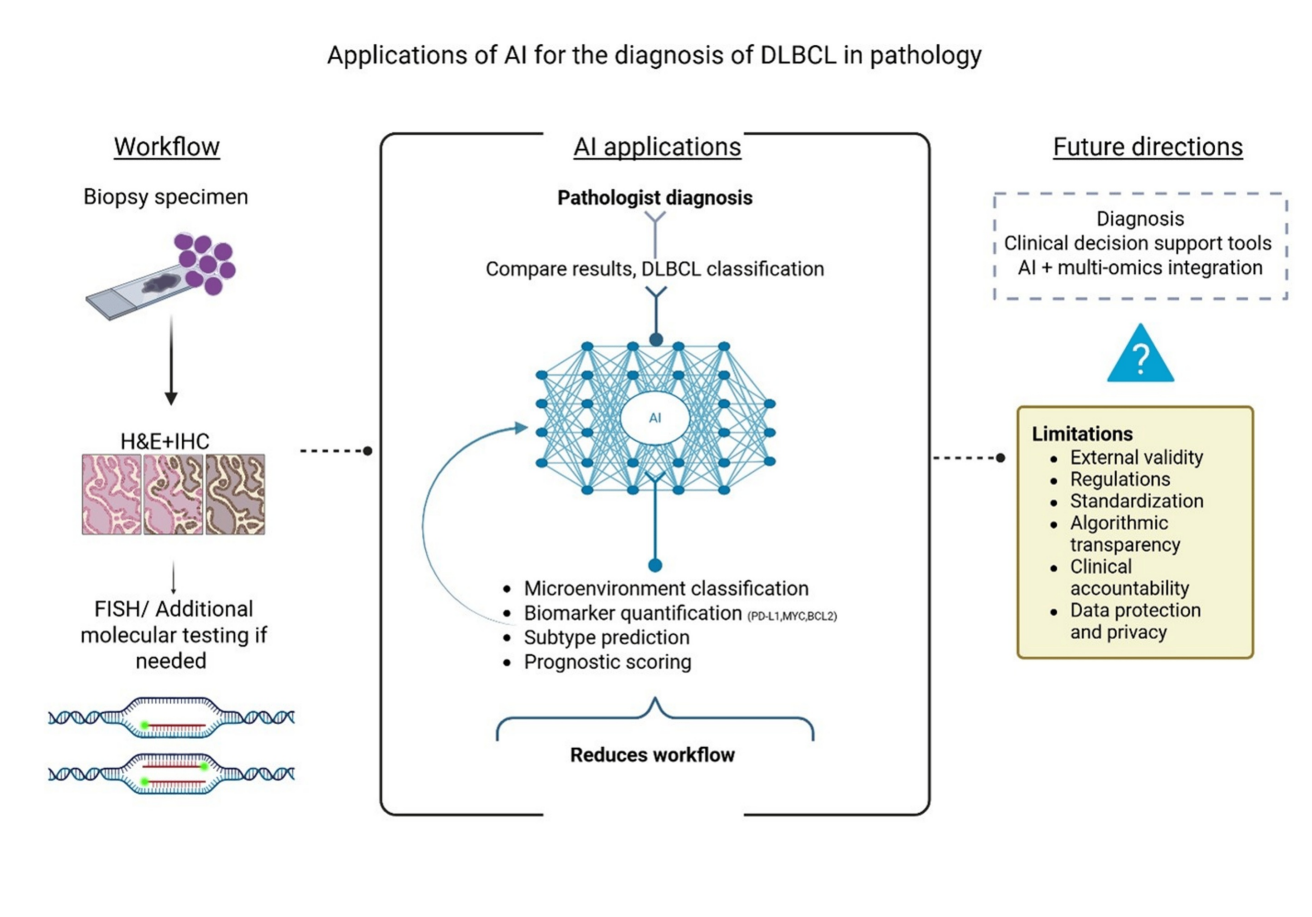
Representation of the application of AI in pathology DLBCL: diffuse large B-cell lymphoma; embedded; FISH: fluorescence in situ hybridization; IHC: immunohistochemistry Created by Jessica D. Torres Luna using BioRender

This holds potential value in both resource-rich and high-volume settings. However, safe and standardized integration into clinical workflows still requires external validation, regulations, and thorough monitoring. The development of explainable AI systems that generate interpretable outputs will be important to increase clinician trust and facilitate adoption. Finally, integrating histopathology with genomic and clinical data, as attempted by Wang et al. through combined histology transcriptomic pipelines, may further improve diagnostic accuracy and support more personalized approaches to DLBCL management. Importantly, these studies highlighted AI’s potential for diagnostics, profiling, and biomarker quantification. They offer insight into how AI could complement pathologists’ expertise and support clinical decision-making. A representation overview of AI application and integration in DLBCL pathology is presented in Figure 4.

Future research should focus on prospective multicenter studies with diverse tissue sources and clinical outcomes. Standardizing metrics and model reporting will help interpretability and reproducibility. Successful implementation of AI will depend not only on performance but also on coordinated teamwork across pathologists, bioinformaticians, clinicians, and regulatory authorities. Partnerships are essential for establishing standardized evaluation, ensuring interpretability of results, and addressing ethical concerns related to algorithmic transparency, confidentiality, data protection, privacy, and clinical accountability [25].

Limitations

While capable, the evidence base is limited. Most studies employed a retrospective study design and used data from single institutions, reflecting whether there was selection bias and limited generalizability. Sample sizes vary widely, and pre-annotated datasets could introduce AI model overfitting risks. Several studies also lacked direct comparisons with full diagnostic workflows, omitting the established routine for DLBCL diagnosis, like IHC or molecular testing, and overplaying AI applications on clinic-based conditions.

## Conclusions

AI remains a critical question in the field of digital pathology. As technological innovation continues to influence the roles of physicians, AI offers tangible benefits by improving diagnostic accuracy and supporting efficient workflows. This systematic review demonstrates that diverse models, ranging from CNNs to ViTs, can achieve high diagnostic performance in DLBCL, in some cases equaling or exceeding pathologist evaluations. However, the real-world application of AI in pathology depends on comprehensive external validation, transparent reporting, and adherence to standardized protocols. Most studies remain retrospective, limited to single institutions, and susceptible to selection bias, highlighting the need for broader, multicenter evidence. AI should be regarded not as a replacement but as an advanced resource that enhances precision, consistency, and efficiency in pathology practice. With collaborative development and rigorous validation, AI has the potential to become a valuable decision-support tool that complements pathologists and contributes meaningfully to improved patient care.

## Appendices

Appendix A 

**Table 4:** Full-text articles excluded at eligibility stage with reasons per the PRISMA guidelines DLBCL: diffuse large B-cell lymphoma; PRISMA: Preferred Reporting Items for Systematic reviews and Meta-Analyses

First author (year)	Title	Reason for exclusion
El Hussein (2022)	Artificial intelligence strategy integrating morphologic and architectural biomarkers provides robust diagnostic accuracy for disease progression in chronic lymphocytic leukemia	Non-DLBCL population; doi: 10.1002/path.5795
Irshaid (2022)	Histopathologic and machine deep learning criteria to predict lymphoma transformation in bone marrow biopsies	Prognostic focus (disease progression and risk prediction). doi: 10.5858/arpa.2020-0510-OA
Basu (2022)	Deep discriminative learning model with calibrated attention map for the automated diagnosis of diffuse large B-cell lymphoma	Full text not retrievable (closed access); doi: 10.1016/j.bspc.2022.103728
Syrykh (2024)	MYC rearrangement prediction from LYSA whole slide images in large B-cell lymphoma: a multicentric validation of self-supervised deep learning models	Full text not retrievable (closed access); doi: 10.1016/j.modpat.2024.100610
